# Geographical origin traceability of sweet cherry (*Prunus avium (L.) Moench*) in China using stable isotope and multi-element analysis with multivariate modeling

**DOI:** 10.1016/j.fochx.2024.101477

**Published:** 2024-05-16

**Authors:** Shuanghui Wang, Piao Chen, Yuchao Liu, Chang Chen, Jing Tian, Zhi Liu, Bin Li, Xianxian Mei, Youlan Chen, Yue Zhang, Chenghao Li, Can Xu, Hansheng Gong

**Affiliations:** a College of Agriculture and Biotechnology, Hunan University of Humanities, Science and Technology, Loudi 417000, China; b Qinghai Light Industry Research Institute Co., Ltd, Xining 810016, China; c Chinese Academy of Inspection and Quarantine, Beijing 100000, China; d School of Food Engineering of Ludong University, Yantai 264000, China; e Changsha Xichu Information Technology Co. LTD, Changsha 417000, China

**Keywords:** Cherry, Stable isotope, Multi-element, Origin traceability, Multivariate analysis

## Abstract

The deliberately origin mislabeling of sweet cherry causes significantly disruptions to market integrity and consumers' trust. In this study, 153 cherry samples from five provinces in China and the corresponding irrigation water and soil samples were collected. 5 stable isotope ratios (δ^13^C, δ^15^N, δ^2^H, δ^18^O, ^87^Sr/^86^Sr) and 8 multi-element contents (Na, Mg, P, K, Ca, Fe, Zn, Se) of cherry were determined by EA-IRMS and ICP-MS to study isotopic fractionation and elemental enrichment mechanisms for origin traceability. The results show the δ^2^H and δ^18^O of cherry exhibit a strong correlated with its irrigation water (r^2^ > 0.85), while δ^15^N, ^87^Sr/^86^Sr, Fe, Zn and Se contents are related to its cultivated soil (r^2^ > 0.75), and the δ^13^C is related to the local microclimate. ANOVA reveals that the regional differences of δ^13^C, δ^2^H, δ^18^O, ^87^Sr/^86^Sr as well as Na, Mg, Ca contents of cherry are significant (*P* < 0.05), and are important geographical indicators. Various multivariate modeling methods, HCA, PLS-DA, and LDA, were employed with the overall accuracy exceeding 90%. This strategy provides an effective mean to verify the label authenticity of cherry origin in Chinese market.

## Highlights

• Stable isotope and multi-element signatures of cherry in China were determined.
• The correlations of cherry with its irrigation water and soil were fully discussed.
• Isotopic and multi-elemental regional variances of cherry in China were compared.
• LDA modeling verified the place of origins of cherry in China with accuracy of 90%.
• This strategy may be a feasible tool for combating the mislabeling of cherry origin.

## 1 Introduction

Cherry, renowned as the “Diamond of fruits” is the fruit of plants belonging to the genus Prunus. It is highly esteemed by consumers for its delightful taste and abundance of health-promoting substances (Ballistreri et al., 2013; Damar & Eksi, 2012; Xu et al., 2021). According to the Food and Agriculture Organization of the United Nations (FAO), China's cherry production reached 3.5 kt in 2020 (FAO, 2020). Currently, various sweet cherry varieties are extensively cultivated in Shandong, Liaoning, and Shaanxi provinces of China. Moreover, cultivation areas are rapidly expanding in other provinces such as Sichuan and Yunnan (Zhang, Yan, Zhang, Wang, & Duan, 2019). Yantai city in Shandong province and Dalian city in Liaoning province have been recognized as historical origins of premium cherries within China. In fact, both Yantai and Dalian big cherries obtained Protected Geographical Indication (PGI) certification in 2007 and 2018 respectively. PGI-certified cherry products can be sold at higher prices with increased market competitiveness compared to non-PGI cherry products (Rabadán, Martínez-Carrasco, Brugarolas, & Bernabéu, 2021). However, with the rapid expansion of cherry planting across China, accurately identifying the geographical origins of cherry products in the domestic market has become a significant challenge. Driven by economic gains, illegal mislabeling practices regarding origin information or selling inferior products under premium cherry labels frequently occur. The aforementioned actions not only undermine market integrity but also hinder the development of China's cherry industry and trade.

Referring to the European Union's proposal of a quality scheme for identifying agricultural products with protected designation of origin (PDO) and protected geographical indication (PGI) (Union, 1992), the Agricultural Department of China also emphasizes the necessity of clearly labeling the geographical indications of agricultural products to ensure their quality and characteristics. (Instrumentalities of the State Council, 2007). In order to comply with these regulations and policies, numerous studies are currently being conducted on traceability and authenticity of agricultural products using various analytical techniques (Wadood, Guo, Zhang, Hussain, & Wei, 2020). To date, very limited research has been reported regarding tracing the origin of cherry products. Maria Vavoura et al., has conducted sensory evaluation and gas chromatography–mass spectrometry (GC–MS) to analyze the volatile compounds and physicochemical parameters of cherry samples, characterized sweet cherry grown in Northern Greece with accuracy exceeding 90% (Vavoura, Badeka, Kontakos, & Kontominas, 2015). Loannis Ganopoulos et al., developed a method utilizing microsatellite genotyping combined with high resolution melting (HRM) analysis to authenticate PDO cherry products from Northern Greece (Ganopoulos, Argiriou, & Tsaftaris, 2011). Matos-Reyes et al., detected element content of Alicante's Mountain cherry for the purposed of origin authentication (Matos-Reyes, Simonot, Lopez-Salazar, Cervera, & Guardia, 2013). Longobardi et al., have successfully applied non-targeted ^1^H nuclear magnetic resonance (NMR) fingerprinting analysis, electronic nose (EN) coupled with isotope ratio mass spectrometry (IRMS) for distinguishing the geographical origin of Italian sweet cherry (Longobardi et al., 2013; Longobardi et al., 2015). Recent years, stable isotope and multi-element analysis have emerged as recognized techniques for tracing the origin of agro-products and successfully applied to various fruits, e.g., durian (Zhou et al., 2021), mango (Muñoz-Redondo et al., 2021), avocado (Muñoz-Redondo et al., 2022), kiwifruit (Xu, 2021), jujube (Wang et al., 2020), and muskmelon (Li B., et al., 2022). There is currently a lack of relevant literature reporting on the application of stable isotope and multi-element analysis in verifying the geographical origins of cherry products from different provinces in China.

The fundamental principles of stable isotope technique for origin traceability of plant-derived agro-products are that the stable isotopic fractionation of agro-product is affected by eco-climate conditions at the place of origin, e.g., temperature, solar irradiance, and precipitation. As a result, these isotopic ratios offer valuable insights into the photosynthesis (carbon fixation), fertilization pattern (nitrogen absorption), precipitation (hydrogen and oxygen), and soil background, thereby serving as geographical indicators for agro-products (Wang et al., 2020). The carbon isotope ratio (δ^13^C) of plant primarily reflects the isotopic fractionation in CO_2_ fixation during photosynthesis in leaf and is affected by climate factors such as solar irradiance, temperature and aridity in the place of origin (Igamberdiev, Mikkelsen, & Gardeström, 2004; Leng & Marshall, 2004). The nitrogen isotope ratio (δ^15^N) of plant mainly depends on fertilization practices and cultivated soil, it manifest the distinctive characteristics about the absorption of inorganic nitrates or ammonium compounds (NO_2_^−^, NH_4_^+^, NO_3_^−^) present in fertilizer and soil (Alison, Bateman, & Timothy, 2005; Craine, Brookshire, Cramer, & Wang, 2015). The hydrogen and oxygen isotopic ratios (δ^2^H and δ^18^O) of plant are dependent on the isotopic compositions of irrigation water and precipitation in the local. According to Global Network of Isotopes in Precipitation (GNIP) published by IAEA, hydrogen and oxygen stable isotopic distribution in rainfall is gradually depleted from coastal region to inland and from flat area to high-altitude region (IAEA/WMO, 2009). The isotopic compositions (^2^H/^1^H, ^18^O/^16^O) of precipitation exhibit regional variations based on the latitudes, altitude, and coastal proximity of origin. The δ^2^H and δ^18^O values contribute to the unique geographical fingerprint of agro-products and can serve as reliable indicators for tracing their origin (Kelly, Heaton, & Hoogewerff, 2005). The strontium isotopes (^87^Sr and ^86^Sr) present in terrestrial plant are sourced from the soil, therefore their spatial variability depends on the regional pedological conditions (McArthur & Howarth, 2005). The bioavailability and root absorption of trace elements are strongly affected by ecological factors in the origin of plant, such as soil pH, temperature, precipitation, aridity, etc., as well as the background levels of heavy metals (e.g., Al, Pb, Ba), which make trace elements unique geographical markers for origin traceability (Wang et al., 2020).

The objectives of the present study were to: (1) investigate the relationships of stable isotope and multi-element signatures between cherry, its irrigation water and cultivated soil; (2) characterize regional variations of stable isotope and multi-element compositions of cherry samples collected from different regions in China; (3) establish multivariate classification and discrimination models to identify the geographic origins of cherry products. This study maybe promising to be as an effective analytical method for combating deliberate mislabeling and fraudulent practices related to the origin of cherry products in the Chinese market, enhancing the reputation of authentic PGI products, and ensuring food safety and authenticity.

## 2 Materials and methods

### 2.1 Chemicals and apparatus

All of reference materials used for stable isotope multi-point calibration provided by International Atom Energy Organization(IAEA, Vienna, Austria): IAEA-CH6 (Sucrose, *δ*^13^C = − 10.5 ± 0.1 ‰), IAEA-600 (Caffeine, *δ*^13^C = − 27.8 ± 0.1 ‰, *δ*^15^N = 1.0 ± 0.2 ‰), B2174 (Urea, *δ*^13^C = − 37.3 ± 0.1 ‰, *δ*^15^N = −0.5 ‰), B2155 (Protein, *δ*^13^C = − 27.0 ± 0.1 ‰, *δ*^15^N = 5.9 ± 0.9 ‰), IAEA N-2 (Ammonium sulfate, *δ*^15^N = 20.3 ± 0.5 ‰), B2203 (IRMS EMA P1, *δ*^18^O = 21.0 ‰, *δ*^2^H = − 25.3 ‰), IAEA-601 (Benzoic acid, *δ*^18^O = 23.3 ± 0.3 ‰), IAEA-602 (Benzoic acid, *δ*^18^O = 71.4 ± 0.5 ‰), and IAEA–CH7 (Polyethylene, *δ*^2^H = − 100.3 ± 2.0 ‰). High purity reference gases, CO_2_ (*δ*^13^C = − 27.92 ± 0.15 ‰, purity *>*99.99%), N_2_ (*δ*^15^N = − 1.3 ± 0.2 ‰, purity *>*99.999%), H_2_ (*δ*^2^H = − 222.6 ± 1.5 ‰, purity *>*99.99%) and CO (*δ*^18^O = 14.6 ± 0.3 ‰, purity *>*99.99%), and He carrier gas (purity *>*99.9999%) for EA-IRMS analysis were purchased from Jingong Special Gas Co., Ltd. (Hangzhou, China). Nitric acid (HNO_3_, GR) and hydrogen peroxide (H_2_O_2_, AR) were purchased from Merck regent company (Germany) for ICP-MS sample digestion. Ultrapure water (18.2 MΩ.cm) was prepared daily via water passed through a Milli-Q water purification system (Bedford, USA). Strontium isotopic standard (strontium carbonate, SRM 987) is obtained from National Institute of Standards and Technology (NIST, Washington, USA) for the calibration of ^87^Sr/^86^Sr value.

Elementar Vario PYRO cube elemental analyzer with Isoprime 100 isotope ratio mass spectrometry system (EA-IRMS, Elementar Analysen systeme GmbH, Germany) was run for four ‘light’ stable isotope ratio analysis. Neptune Plus multiple-collector inductively coupled plasma mass spectrometry system (MC-ICP-MS, Finnigan Mat GmbH, Germany) was conducted for ‘heavy’ stable isotope ratio analysis, i.e., ^87^Sr/^86^Sr. Thermo Fisher X-series II inductively coupled plasma mass spectrometry system (ICP-MS, Thermo Co. Ltd., USA) was conducted for multi-element analysis. MARSX microwave digestion oven equipped with a 12-position carousel and 6 Teflon® XP-1500 Plus high-pressure vessels (CEM Corp., Milan, Italy) was run for sample digestion. JXFSTPRP-1152 liquid nitrogen grinder (Jingxin™, China), a FD vacuum lyophilizer (Shunzhi™, China) and a mini homogenizer (Joyoung™, China) were used for sample pretreatment. MS105DU millionth balance and MS204S ten thousandth balance (Mettler Toledo, Switzerland) were used for sample weighing.

### 2.2 Sampling and pretreatment

**Fig. 1:**
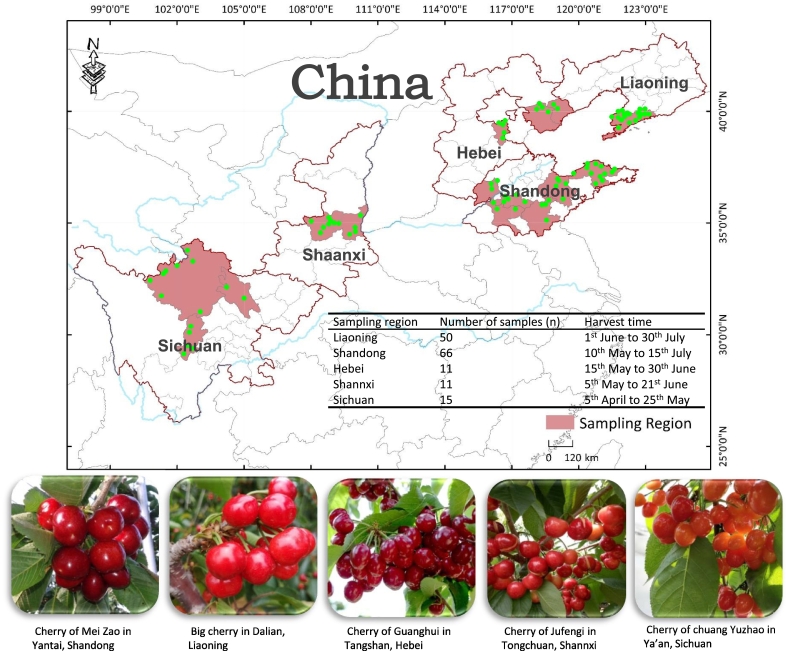
The agricultural geographical information system (ArcGIS) distribution plot of sampling sites of 153 cherry orchards in five provinces of China (Liaoning, Shandong, Hebei, Shannxi, Sichuan).

Representative sampling is deemed as a crucial step for ensuring the reliability and stability of origin traceability of agro-products. In this work, total of 153 cherry samples were collected from the main producing areas distributed in five different provinces of China (Liaoning, Shandong, Hebei, Shannxi and Sichuan) during the harvest time from April to July in 2019 and 2020. To ensure the authenticity of the place of origin of cherry sample, every sample were directly collected by our team member from local cherry orchard, and no repetition in each orchard. The specific sampling number in different provinces was planed according the scale of cherry farming in local. The sampling number was 50 for Liaoning (S1-S50), 66 for Shandong (S51-S116), 11 for Hebei (S117-S127), 11 for Shannxi (S128-S138) and 15 for Sichuan (S139-S153), respectively. The corresponding irrigation water and soil around root (20 cm to 40 cm) were collected from cherry orchards, packaged or bottled, and then transported to the laboratory for analysis. The geographical distribution information of sampling sites was recorded using Agricultural Geographic Information System (ArcGIS) (as shown in Fig. 1). the specific sampling processes of cherry, irrigation water and cultivated soil were carried out as follows:

• *Cherry:* in each orchard, at least 5 kg of cherry sample was collected at three sampling sites distributed along the diagonal of each orchard. The cherry fruit must exhibit a maturity level with sweetness exceeding 13, absence of ulceration or lesions, and a size larger than 28 mm, which reaches JJ or 10 ROW referring to international standard (as pictures shown in Fig. 1). Notably, the distance between each two sampling orchards must be at least 5 km away. In the laboratory, cherries was removed their stone using an automated pitting machine. Then, 200 g cherry flesh was homogenized and the resulting sample was frozen at −20 °C for 12 h. Thirdly, the frozen sample was pulverized into fine ice particles using a micromill and transferred to the lyophilizer chamber for vacuum freeze-drying process at −80 °C and − 15 MPa for 48 h. Finally, the fully freeze-dried sample was ground in a liquid nitrogen grinder and passed through a 100-mesh sieve, fine powder was collected and stored sealed in brown vial at −80 °C before stable isotope analysis. In addition, the remaining cherry flesh sample was also homogenized and rapidly frozen at −80 °C for Sr stable isotope analysis and multi-elemental determination.
• *Irrigation water:* in the vast majority of cherry orchards of China, irrigation water is primarily sourced from local river or reservoir and introduced into the orchards through pipelines during the young fruit stage of cherry. Therefore, water samples collected from these local rivers or reservoirs were used as representative samples of irrigation water. A total of 2000 mL water was collected at three sampling sites along river or reservoir and stored in a brown plastic bottle with a lid. It is important that water must be fully filled without any significant empty to avoid hydrogen and oxygen fractionation effects caused by water evaporation. The bottled water sample was then transported to the laboratory at 4 °C to minimize the microbial activities. In the laboratory, the water sample was further filtered using a 0.22 μm fibrous membrane filter to remove the microorganisms and organic impurities. Finally, the filtered water sample was injected into a sample vial for stable isotope analysis. It should be noted that sample vial should be fully filled without any cavity in the whole analytical procedure.
• *Cultivated soil:* at three sampling sites of cherry orchard, 0–40 cm root soil layer surrounding cherry tree was collected by an auger boring and mixed together, at least 10 kg of soil was delivered to laboratory. In laboratory, soil was tiled on a specimen disc at 2 cm thickness and fully air-dried at room temperature. Then, the dried soil was further subdivided by quartering to around 500 g, ground and passed through 100-mesh sieve, fine powder was collected for the representative sample of cultivated soil.

Therefore, total of 153 freeze-dried cherry flesh samples, 153 homogenized cherry flesh samples, 153 irrigation water samples and 153 cultivated soil samples were collected and prepared in this work.

### 2.3 Stable isotope ratio (SIR) analysis

Fine powdered samples of cherry and soil were weighed in triplicate into 4 × 11 mm tin capsules and 4 × 11 mm silver capsules (approximately 2.00 mg for the δ^13^C and δ^15^N analysis, 1.50 mg for the δ^2^H and δ^18^O analysis of cherry sample, approximately 1.00 mg for the δ^13^C and δ^15^N analysis of soil), the packaged sample must be equilibrated for 3 days in a vacuum desiccator for EA-IRMS analysis. All vails of water samples were put on the automatic sampling tray of EA in detection sequence and injected 10 μL into EA oven by a sampling needle for δ^2^H and δ^18^O analysis. In C/N mode, EA oxidation and reduction furnace temperatures were respectively set at 920 °C and 600 °C, high-purity (> 99.999%) CO_2_ (δ^13^C = −27.92 ‰) and N_2_ (δ^15^N = −1.3 ‰) served as reference gases. In H/O mode, EA pyrolysis was undertaken at 1450 °C, high-purity (> 99.99%) H_2_ (δ^2^H = −222.6 ‰) and CO (δ^18^O = +14.6 ‰) served as reference gas. Helium gas (purity >99.9999%) at flow rate of 230 mL min^−1^ was used as carrier gas. Stable isotope ratios were calculated via Eq. (1) referring to the literature published by Coplen (Coplen, 2011):

(1) $$\delta \left(‰\right)=\frac{R_{\mathrm{sample}}}{R_{\mathrm{standard}}}-1$$

where *δ* represents either *δ*^13^C, *δ*^15^N, *δ*^2^H or *δ*^18^O; R_sample_ denotes the abundance ratio of heavy isotope against light isotope, e.g., ^13^C/^12^C, ^15^N/^14^N, ^2^H/^1^H, ^18^O/^16^O; R_standard_ is the isotope ratio of reference standard versus primary standards, Pee Dee Belemnite (VPDB) for carbon, Air gas (AIR) for nitrogen, Standard Mean Ocean Water (VSMOW) for hydrogen and oxygen. In this study, IRMS reference standards were purchased from IAEA (International Atomic Energy Agency, Vienna) and used as QC samples for the multi-point isotopic calibration: IAEA–CH6, IAEA-600, B2174 and B2155 for *δ*^13^C, B2155, B2174, IAEA-600, IAEAN-2 for *δ*^15^N, IAEA–CH7, B2203 for *δ*^2^H, B2203, IAEA-601, IAEA-602 for *δ*^18^O, respectively. Instrumental precision and reproducibility (represented as RSDs) were lower than ±0.2 ‰, ± 0.2 ‰, ± 1.5 ‰ and ± 0.3 ‰ for *δ*^13^C, *δ*^15^N, *δ*^2^H, *δ*^18^O, respectively.

### 2.4 Sr isotope analysis

The strontium isotopic ratios (^87^Sr/^86^Sr) of cherry and cultivated soil samples were measured using MC-ICP-MS. About 0.5 g of cherry and soil fine powder samples were weighted and digested by microwave (MW) assisted acid digestion step. First, cherry and soil samples were reacted with 5.0 mL ultrapure HNO_3_ for at least 2 h. Afterwards, the sample solutions were transferred into the digestion vessels, and the mineralization were carried out. Then, the mineralization of sample was at 150 °C for 20 min of duration time to completely digest all residual organic matters. Next, the solubilization of sample was accomplished by MARSX microwave oven equipped with a temperature probe (RTP-300 Plus) and pressure sensor (ESP-1500 Plus) for temperature and pressure monitoring on the reference vessel. Each mineralization batch consists of five real samples and one blank sample composed of 10 mL ultrapure HNO_3_. Finally, sample solutions were transferred into perfluoroalkyl (PFA) resin bottles and added an appropriate amount of ultrapure HNO_3_ to obtain final solutions. The data of ^87^Sr and ^86^Sr contents of sample were acquired using 10 blocks of 20 cycles in 4.2 s integration time with 6 s idle time between magnet jumps. Experimental conditions and interference correction were then applied according to the general program (listed in **Table S1 in supporting information**). The measured value of strontium isotopic ratio (i.e., ^87^Sr/^86^Sr) of sample was the average value of the results after 70 repetitions. The calibration of ^87^Sr/^86^Sr of sample was as follow referring to the equation reported by Konter & Storm, et. al. (Konter & Storm, 2014): ^87^Sr/^86^Sr = ^87^Sr/^86^Sr_sample_ × (0.710235/^87^Sr/^86^Sr of SRM987), the precision (represented by RSD%) is lower than 0.01%, and Rb/Sr < 0.01.

### 2.5 Multi-element analysis

Multi-elemental contents of cherry and cultivated soil samples were measured by ICP-MS. 1.5000 g of cherry flesh homogenized sample and 50.00 mg powdered soil sample were accurately weighted in triplicate and digested in 60 mL acid-washed microwave oven vessels containing 5.0 mL HNO_3_ (GR, Sigma-Aldrich, Shanghai) and heated to 60 °C in a microwave digestion system (Milestone Ethos one, ITA) under temperature control mode for 40 min. After cooling, 1.0 mL of H_2_O_2_ (AR, Sigma-Aldrich, Shanghai) was added into the vessels, and then they were placed on a graphite heater at 160 °C to evaporate the acid until the digestion liquid volume was reduced to around 1 mL. Cooled digested liquids were subsequently diluted to 25.0 mL with deionized water for ICP-MS analysis. A mixed internal standard solution (10 ng mL^−1^) of Y (GSB04–1788-2004), Sc (GSB04–1757-2004), Ge (GSB 04–1728-2004), Rh (GSB 04–1746-2004) and Re (GSB04–1745-2004) were prepared using their standard solutions of 100 ng mL^−1^ purchased from National Center of Analysis and Testing for Nonferrous Metals and Electronic Materials (Beijing, China) for checking instrumental drift. A standard solution containing all 8 analytical elements (Na, Mg, P, K, Ca, Fe, Zn, Se) was obtained from Sigma-Aldrich and diluted into five aliquots of different concentrations with ultra-pure water for multi-point calibration. The limits of detection (LODs) for eight elements detected in cherry and soil: Na, Mg, P, K, Ca, Fe, Zn, Se. were 1, 1, 1, 1, 1, 1, 0.5 and 0.01 mg kg^−1^, the limits of quantitation (LOQs) were 3, 3, 3, 3, 3, 3, 1.5 and 0.05 mg kg^−1^ respectively.

### 2.6 Statistic and software

One-way analysis of variance (ANOVA) was initially conducted to assess the significance level of each variable between groups and its impact on classification (St & Wold, 1989). However, it is often challenging to fully differentiate similar groups using a single variable. Therefore, multivariate modeling methods such as Hierarchical Cluster Analysis (HCA), Partial Least Squares Discriminant Analysis (PLS-DA), and Linear Discriminant Analysis (LDA) were employed to extract discriminant information from all variables for clearer group classification. Unsupervised HCA was utilized for dimensionality reduction and data denoising by calculating the Mahalanobis Distance between every pair of samples and clustering them based on nearest neighbor principle (Bridges Jr, 1966). Furthermore, transitioning from unsupervised modeling to supervised modeling with PLS-DA and LDA aimed at achieving more satisfactory discriminant results for samples. Supervised PLS-DA and LDA utilize prior information about sample classes to enable more accurate discrimination. Based on this prior information, PLS-DA decomposes and fits observed or measured characteristic variables in order to search for a linear regression model that projects observed variables onto predicted variables in a new space for sample classification purposes (Ballabio & Consonni, 2013). On the other hand, LDA constructs new linear discriminant functions by simultaneously minimizing within-group variance while maximizing between-class variance in order to enhance discriminant performance. This reduces the dimensionality of data points onto the same line while keeping them as separated as possible (Ioffe, 2006). Finally, leave-one-out cross validation is performed to verify the accuracy of the established PLS-DA and LDA models, sample test is performed on random 2000 cycles. One of prominent advantages of leave-one-out cross-validation is that the largest possible number of samples can be used for training in each iteration and that each calculate results is certain (the result of each execution is consistent) (Browne, 2000).

ANOVA, LDA modeling, receiver operating characteristic curve (ROC) test of variable were conducted on SPSS software (ver.19.0, IBM, USA), HCA heat map and boxplot were plotted by R language (ver. 4, Otago university), PLS-DA modeling was performed on SICMA-p (ver. 10, Umetrics, Sweden).

## 3 Results and discussion

### 3.1 Relationships of cherry with irrigation water and cultivated soil

**Fig. 2:**
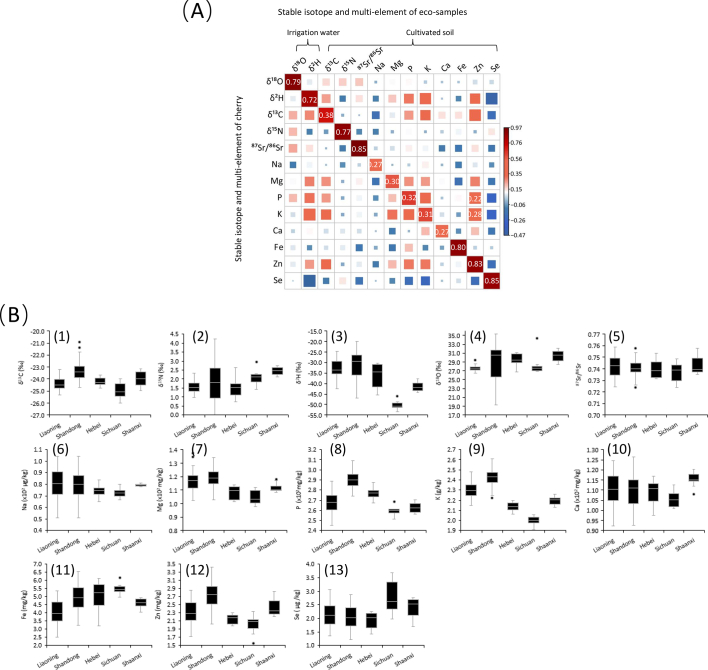
(A) The correlation heat map of isotopic and multi-elemental signatures of cherry with its irrigation water and cultivated soil. (B) the boxplots of five stable isotope ratios and eight multi-elemental contents of 153 cherry samples from five provinces of China (Liaoning, Shandong, Hebei, Shannxi, Sichuan).

Analytical results of mean value and standard deviation of five stable isotope ratios (δ^13^C, δ^15^N, δ^2^H, δ^18^O and ^87^Sr/^86^Sr) and eight elemental content of cherry samples and their corresponding irrigation water and cultivated soil collected from five different provinces of China (Liaoning, Shandong, Hebei, Sichuan, Shannxi) were summarized in Table 1. The multivariate correlation analysis (presented as heat map) of stable isotope and multi-element signatures of cherry samples with the corresponding irrigation water and cultivated soil samples were conducted (see Fig. 2A), which comprehensively visualizes the correlations of variables and reveal their migration mechanism from irrigation water or cultivated soil to cherry.

**Table 1:** The mean values, standard deviations and ANOVA results of stable isotope ratios and elemental contents of 153 cherry samples, the corresponding irrigation water and cultivated soil samples from five provinces of China (Liaoning, Shandong, Hebei, Shaanxi, Sichuan).

Variable	Liaoning (*n* = 50)		Shandong (*n* = 66)		Hebei (*n* = 11)		Shaanxi (n = 11)		Sichuan (*n* = 15)	
	Soil/Water	Cherry	Soil/Water	Cherry	Soil/Water	Cherry	Soil/Water	Cherry	Soil/Water	Cherry
δ^13^C [‰]	−23.0±0.7	−24.4±0.5^b^	−22.2±1.1	−23.4±0.8^a^	−22.9±0.6	−24.2±0.3^b^	−22.8±1.3	−24.0±0.6^b^	−23.4±0.9	−25.0±0.6^c^
δ^15^N [‰]	1.2±0.4	1.6 ± 0.3^b^	1.4±0.9	1.8±1.1^b^	1.1±0.3	1.5±0.5^b^	1.9±0.4	2.4±0.2^a^	1.7±0.5	2.1±0.5^a^
δ^2^H [‰]#	−33.3±5.8	−33.0±4.2^b^	−31.5±8.5	−31.0±6.5^a^	−34.2±9.6	−36.2±5.7^b^	−42.2±4.97	−41.5±2.2^d^	−50.1±7.8	−50.4±1.8^c^
δ^18^O [‰]#	30.4±2.3	30.5±1.2^a^	29.2±4.4	28.9±3.9^ab^	29.9±1.8	29.5±1.2^ab^	27.2±1.2	27.6±0.7^b^	27.9±1.9	28.0±1.8^b^
^87^Sr/^86^Sr	0.71±0.02	0.71±0.01^b^	0.74±0.01	0.71±0.03^b^	0.74±0.02	0.71±0.01^b^	0.74±0.03	0.72±0.03^b^	0.81±0.04	0.75±0.02^a^
Na [μg kg^−1^]	1629.2±524.6	79.9±12.5^a^	1510.0±519.6	79.7±12.5^a^	1520.0±238.1	74.5±4.9^ab^	1387.5±358.7	79.4±1.0^ab^	1703.9±324.2	72.6±3.8^b^
Mg [mg kg^−1^]	3588.9±447.2	117.0±7.6^a^	3585.1±480.3	118.6±7.0^a^	3211.6±399.3	108.7±4.5^bc^	3265.1±247.0	111.7±3.1^b^	3075.6±447.9	104.5±4.9^c^
P [mg kg^−1^]	2606.9±210.8	267.1±10.9^c^	2934.0±225.4	290.0±7.4^a^	2703.5±159.7	277.0±5.6^b^	2629.8±218.2	262.9±4.5^cd^	2580.6±245.5	259.9±5.5^d^
K [g kg^−1^]	223.0±2.2	2.3±0.1^b^	24.2±2.3	2.4±0.1^a^	22.0±2.1	2.1±0.0^c^	22.1±2.2	2.2±0.0^c^	19.2±2.5	2.0±0.0^d^
Ca [mg kg^−1^]	537.7±86.7	110.4±8.2^ab^	533.5±101.9	109.7±8.4^b^	533.7±115.6	109.2±5.7^b^	569.0±88.0	115.4±3.4^a^	512.9±76.3	105.2±4.0^c^
Fe [mg kg^−1^]	39,784.2±9336.3	4.1±0.8^c^	49,694.8±8782.4	5.0±0.8^b^	51,749.5±9995.1	5.0±0.8^ab^	44,087.1±5071.6	4.6±0.3^b^	54,219.1±4904.2	5.5±0.3^a^
Zn [mg kg^−1^]	91.7±16.5	2.3±0.3^bc^	109.0±16.1	2.7±0.3^a^	84.9±8.9	2.2±0.1^c^	96.1±10.0	2.4±0.2^b^	77.6±7.5	2.0±0.2^c^
Se [μg kg^−1^]	86.1±18.7	2.1±0.4^c^	82.8±17.3	2.1±0.4^c^	73.4±14.1	1.9±0.3^c^	101.5±12.1	2.4±0.3^b^	112.8±24.1	2.8±0.6^a^

Note: different lowercase superscripts (e.g., a, b, c) represent the difference of variable between groups are significant at the confident level of 0.05.

# : δ^2^H and δ^18^O of the corresponding irrigation water.

As shown in Table 1 and Fig. 2A, the carbon isotope ratio (δ^13^C) of cherry is poorly correlated with the value of its cultivated soil (r^2^ = 0.38), despite their δ^13^C values being very close (−23.4 ‰ to −22.2 ‰ for soil, −25.0 ‰ to −23.45 ‰ for cherry). This discrepancy can be attributed to the fact that the carbon isotopes of various carbohydrates in cherry fruit (glucose, fructose, sucrose, etc.) primarily originate from atmospheric CO_2_ through the carbon fixation of leaf photosynthesis rather than from carbon-containing gases (e.g., CO_2_ or CH_4_) emitted by soil microbes and inorganic carbonates (CO_3_^2−^ and HCO_3_^−^) present in the cultivated orchard's soil. The δ^13^C values of cherry fruit reflect its absorption of atmospheric CO_2_ and photosynthetic activities of leaf, which are easily influenced by climatic factors such as solar intensity, temperature, and aridity.

The presence of significant positive correlations between δ^15^N values of cherry and cultivated soil is evident (r^2^ = 0.72). The δ^15^N values of soil samples from Sichuan (1.9 ± 0.4 ‰) and Shannxi (1.7 ± 0.5 ‰) are notably higher compared to soil samples from other provinces (approximately 1.1‰). Consequently, the δ^15^N values of cherry in these two provinces exhibit an elevation, which can be attributed to the fact that nitrogen isotopes (^14^N and ^15^N) in cherries primarily originate from nitrogenous nutrients present in cultivated soil, specifically inorganic nitrate (NO_3_^−^, NO_2_^−^) and ammonium compounds (NH_4_^+^). The nitrogen isotopic compositions (^15^N/^14^N) of soil in different production areas are closely associated with fertilization patterns in conventional or organic farming, which is influenced by the local orchard's soil fertility.

The mean δ^2^H and δ^18^O values of irrigation water samples from different sampling sites were in the range of −50.1 to −31.5 ‰ and 27.2 to 30.4 ‰, respectively. It is evident that the spatial distribution of hydrogen and oxygen isotopes in irrigation water shows a depletion trend of heavy isotope from the coastal regions (Liaoning, Shandong, and Hebei) to inland regions (Shannxi and Sichuan), which attributes to natural isotopic fractionation during the atmospheric water cycle in global. In general, the δ^2^H and δ^18^O values of cherry are respectively in the range of 50.4 ‰ to −31.0 ‰ and 27.6 ‰ to 30.5 ‰ across five provinces, which exhibit similar range and regional variations as local irrigation water. Notably, there is a strong positive correlation between the isotopic compositions of cherry and irrigation water, their correlation coefficients (r^2^) are 0.72 for δ^2^H and 0.79 for δ^18^O. These significant correlations between the hydrogen and oxygen isotopes found in organic matters of cherry (such as various carbohydrates) can be attributed to their sources from irrigation water in local orchard despite oxygen is via the indirect pathway. Therefore, the naturel spatial distribution of hydrogen and oxygen isotopic compositions in precipitation is influenced by geographical factors such as latitude, altitude, coastal proximity, making δ^2^H and δ^18^O values of cherry be regional different.

The bioavailability of various mineral elements in soil to plant is affected not only by its texture and properties (e.g., pH, organic matter), agricultural practices (e.g., fertilization and irrigation), but also depends on local microclimate factors such as precipitation, temperature, solar intensity. Correlation analysis indicates that the relationships of various elemental contents between cherry and its cultivated soil, particularly Fe, Zn, and Se, are highly close, which is due to their root intake is direct from soil. The correlation coefficients (r^2^) are 0.80, 0.83 and 0.85, respectively. But Na, Mg, P, K, and Ca contents of cherry show relatively low correlation with its cultivated soil (r^2^ = 0.27, 0.30, 0.32, 0.31), indicating that other sources (e.g., irrigation water or water-soluble fertilizers) besides the soil itself can introduce additional amounts for cherry growth. Furthermore, the heat map reveals a weak synergistic effect between P and K contents of cherry with Zn in soil (r^2^ = 0.28 and 0.22), suggesting that their interaction influences Zn content in cherry.

In general, δ^2^H, δ^18^O, δ^15^N, ^87^Sr/^86^Sr, Fe, Zn, and Se contents of cherry exhibit strong correlations with the backgrounds of its irrigation water or cultivated soil. However, there are relatively weak or even negative correlations observed between their δ^13^C, Na, Mg, P, K, and Ca contents. Further explanations regarding these correlations with the local eco-system are necessary by conducting correlation analysis of stable isotope ratios and multi-element contents between cherry and its irrigation water or cultivated soil using a heat map presentation method, which preliminarily explain the formation mechanism behind regional differences of stable isotopic and multi-elemental signatures. This understanding is crucial for tracing the origin of cherry in China.

### 3.2 Regional variances of stable isotope and multi-elemental signatures of cherry

The ANOVA comparisons (represented as different lowercase superscripts) of stable isotopic ratios and multi-elemental contents of cherry between five origins were summarized in Table 1, and the boxplots of variables further visually presented their regional differences, as shown in Fig. 2B.

The mean δ^13^C values of cherry samples from five provinces of China are generally similar in the ranged from −25.0 ‰ to −23.4 ‰ (see Table 1), the result of ANOVA comparison of δ^13^C indicates that there are significant differences between five provinces (represented by different lowercase superscripts). As shown in Fig. 2**B**-1, the mean δ^13^C value of cherry samples in Shandong is −23.4 ± 0.8 ‰, significantly more positive relative to samples in other provinces, but it is the most negative in Sichuan. The climate in Shandong is warmer and more humid, enhancing the utilization activities of ‘heavy’ isotopic carbon dioxide (i.e., ^13^CO_2_) in leaf photosynthesis. Consequently, organic matters in cherry such as glucose and fructose enriched with ^13^C, leading to an elevation of mean δ^13^C value specifically observed in Shandong relative to other regions. The regional variance of δ^13^C value of cherry is caused by the carbon isotopic fractionation effects during the photosynthesis of various organic matters in leaf, which is dependent on local microclimate (temperature, moisture, solar intensity, etc.) and can serves as an important geographical indicator regarding the place of origin of cherry to some extent.

The overall δ^15^N values of cherry samples from different provinces varied in the range of 1.5 ‰ to 2.4 ‰. Comparatively, the δ^15^N values of cherry samples from Sichuan (2.1 ± 0.5 ‰) and Shannxi (2.4 ± 0.2 ‰) were significantly higher than those of samples from Liaoning (1.2 ± 0.4 ‰), Shandong (1.4 ± 0.9 ‰) and Hebei (1.5 ± 0.5 ‰) (see Table 1). The δ^15^N values of cherry are dependent on the nitrogen isotopic compositions of cultivation soil and agricultural fertilizers used in their production processes. As shown in Fig. 2**B**-2, the higher δ^15^N values observed in cherry from Sichuan, which can be attributed to fertile ‘purple soil’ there. In Sichuan, there is world-famous fertile ‘purple soil’ to provide abundant of organic nitrogen nutrients that contribute to elevating the δ^15^N value of cherry (Li et al., 2022). Similarly, it is likely that organic farming practices with limited use of synthetic nitrogen fertilizers explain the higher δ^15^N value observed in cherry from Shaanxi. Conversely, lower δ^15^N values were found in cherries produced in Shandong, Liaoning, and Hebei due to a greater reliance on synthetic fertilizers such as nitrates and urea being applied extensively in orchards to increase cherry yields. Overall, δ^15^N value of cherry can serve as an indicator reflecting local soil fertility levels to some extent as well as traditional agricultural patterns adopted within local cherry orchards – whether conventional or organic – thus potentially providing insights into their place of origin.

**Fig. 3:**
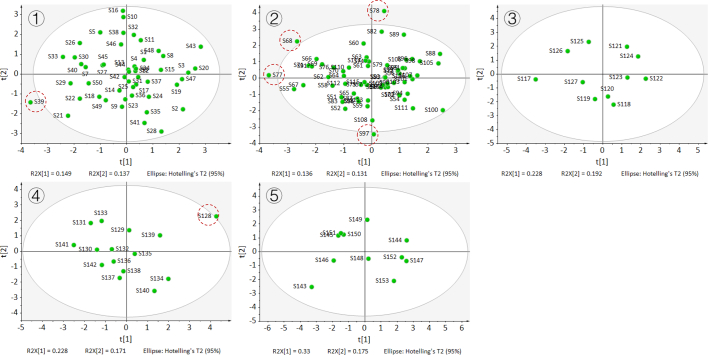
The results of outlier test (at 95% confident degree level) of cherry samples from five province using PCA cluster with 95% confident ellipse: 1) Liaoning; 2) Shandong; 3) Hebei; 4) Sichuan; 5) Shannxi.

**Fig. 4:**
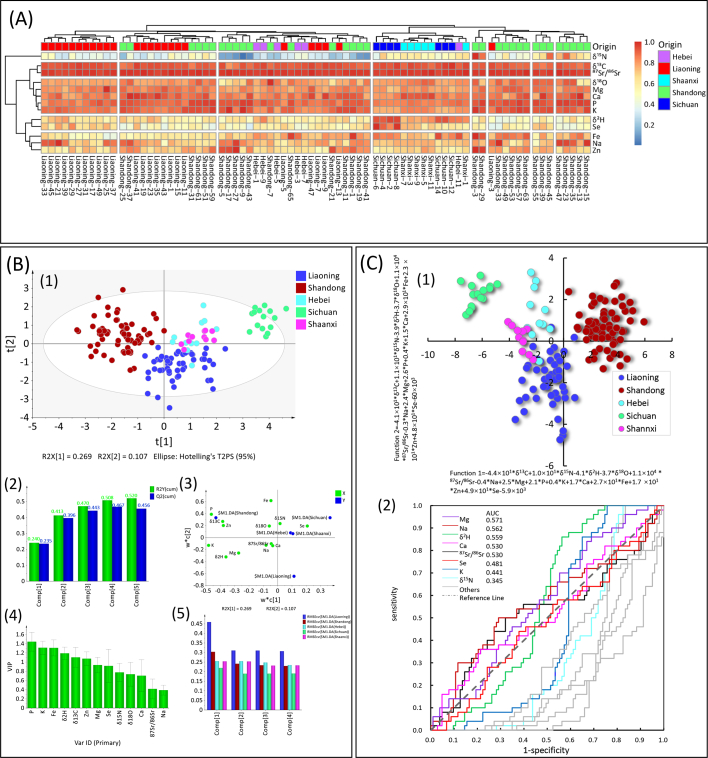
(A) HCA heat map of cherry samples from different provinces of China and classification variables. (B) PLS-DA modeling results: (1) Score plot of 153 cherry samples projected on the first two principal components (t1 and t2); (2) Summary fitting plot of five principal components; (3) Loading plot of variables; (4) VIP plot of variables; (5) Relative mean standard error of first four principal components. (C) LDA modeling results: (1) Score plot of 153 cherry samples projected on the first two discriminant functions (Function 1 and Function 2); (2) ROC and AUC curve of variable.

The δ^2^H values of cherry samples in different provinces are distributed in a large variation range from −50.4 ‰ to −31.0 ‰. The mean δ^2^H values of cherry samples from Sichuan (−50.4 ± 1.8 ‰) and Shannxi (−41.5 ± 2.2 ‰) are obviously more negative than the values of samples from Liaoning (−33.0 ± 4.2 ‰), Shandong (−31.0 ± 6.5 ‰), Hebei (−36.2 ± 5.7 ‰) province, as shown in Fig. 2**B**-3. The ANOVA comparison indicate that the regional differences of δ^2^H values between provinces are highly significant (represented by different lowercase superscripts). The mean δ^18^O values of cherry samples present a basically consistent spatial distribution trend with δ^2^H values across five provinces, 30.5 ± 1.2‰, 28.9 ± 3.9‰, 29.5 ± 1.2‰, 27.6 ± 0.7‰ in Liaoning, Shandong, Hebei, Shannxi, and the lowest being in Sichuan (28.0 ± 1.8‰). According to the principal of GNIP (IAEA, 2008), the rainfalls in Liaoning, Shandong and Hebei is less depleted in ‘heavy’ isotopes (^2^H and ^18^O) due to the proximities of cherry-producing areas to the ocean compared to inland regions in Shannxi and Sichuan, thereby elevating the δ^2^H and δ^18^O values of cherry. However, the regional differences of δ^18^O values between provinces are obviously less significant compared to those of δ^2^H, as shown in Fig. 2**B**-4. Referring to previous literatures (Augusti & Schleucher, 2007; Penna, Geris, Hopp, & Scandellari, 2020), the spatial distributions of δ^2^H and δ^18^O values of plants are reported to be fully dependent on the isotopic compositions of its irrigation water and present similar tends of regional variations. However, the biological pathways for hydrogen and oxygen isotopes incorporated from eco-environment into cherry organic matters through leaf photosynthesis are different. Hydrogen stable isotopes (^1^H,^2^H) directly originate from H_2_O molecule of irrigation water but oxygen stable isotopes (^16^O, ^18^O) are from atmosphere CO_2_ molecule (Newton, 2010). Therefore, the significance of their regional variations is not entirely consistent although oxygen of CO_2_ can exchange with H_2_O in other naturally biochemical cycles. δ^2^H and δ^18^O values can serve as crucial indicators for tracing the origin of cherry by reflecting both spatial distributions of isotopic compositions in local water system and the dynamic changes of water utilization in plant caused by local microclimate factors such as temperature, moisture, solar intensity.

The mean ^87^Sr/^86^Sr ratio values of cherry samples in different provinces of China are all fluctuates around 0.71, just the value of cherry samples is obviously higher in Sichuan (0.75 ± 0.02), as shown in Fig. 2B-5. ANOVA comparison indicates that significant regional difference exists between Sichuan and other provinces (represented by different lowercase superscripts). The ore deposits rich in strontium isotopes (e.g., celestite (SrSO₄), strontianite (SrCO₃)) may be consistent in different provinces of China, but the bio-availability of Sr isotopes is significantly affected by soil texture and properties, e.g., weathering age, air permeability, and pH value. On the one hand, the texture of fertile ‘purple soil’ in Sichuan province is different from poor yellow brown soil in the northern provinces of China, the percentage of humus in the former is significantly higher than the latter. On the other hand, pH value of soil in Sichuan is lower than 7.0 due to the acidification of humus in it, activates the ionization of Sr isotopes and improves the bio-availability of ^87^Sr by cherry tree root, which elevates the ^87^Sr/^86^Sr ratio of cherry in local. ^87^Sr/^86^Sr ratio values of cherry also can be as an indicator for inferring the place of origin of cherry in China.

Multi-elemental fingerprints of cherry samples in different provinces of China were affected by the all-around regional differences of soil texture and properties, farming practices and microclimate together. From the boxplots of element contents in Fig. 2B(6–13), Mg, P, K, Zn, Se of cherry exhibit more significant differences between five provinces of China than Na, Ca, and Fe just present differences in some provinces (represented by different lowercase superscripts). The Mg contents of cherry samples are in the range from 104.5 to 118.6 mg kg^−1^, the mean value in Sichuan is 104.5 ± 4.9 mg kg^−1^, it is relatively higher in Liaoning (117.0 ± 7.6 mg kg^−1^), Shandong (118.6 ± 7.0 mg kg^−1^) and Shannxi (111.7 ± 3.1 mg kg^−1^). Similarly, the mean P, K, Ca, Zn contents of cherry samples in Sichuan are respectively 259.9 ± 5.5 mg kg^−1^, 2.0 ± 0.0 g kg^−1^, 105.2 ± 4.0 mg kg^−1^, 2.0 ± 0.2 mg kg^−1^, and these values are also at lowest levels relative to those in other provinces, which is due to there is less input of potash fertilizer (e.g., KNO_3_ or KCl), phosphate fertilizer (e.g., Ca(HPO_4_)_2_) or Zn-containing fertilizer being used in the fertile ‘purple soil’ in Sichuan for raising the production of cherry. Conversely, Fe element of cherry samples are more abundant in Sichuan (5.5 ± 0.3 mg kg^−1^) relative to other provinces where the mean values are lower than 5.0 mg kg^−1^, the lower pH in purple soil activates the bio-availability of Fe for the absorption of cherry tree root. For Se element of cherry samples, the mean value is 2.8 ± 0.6 mg kg^−1^ in Sichuan, it is also at a obvious higher levels compared to that of other provinces (about 2.1 mg kg^−1^), the producing areas of cherry in Sichuan are located at the distribution region of Se-rich soil in ‘purple soil’. Multi-elemental contents of cherry samples from Shannxi are always in the intermediate levels, of which Na, Mg, P, K, Ca, Zn contents of cherry samples in Shannxi are lower than samples from three provinces along the coast (Shandong, Hebei and Liaoning) but higher than samples from Sichuan, but Fe and Se contents are of reverse variation trends.

The boxplots of stable isotopic and elemental variable between five provinces of China visually presents their regional variances. The overall ranges of δ^13^C, δ^15^N, ^87^Sr/^86^Sr values, and Ca, Na, Se contents of cherry samples from different provinces are overlapped, just the δ^2^H values of cherry samples from Sichuan and K contents of cherry samples from Hebei, Sichuan, and Shannxi can reluctantly be distinguished from samples from other origins. In this context, no matter single isotopic variable or elemental variable, it is difficult to distinguish the origin of cherry from all provinces, multivariate statistical methods were needed to extract the important difference information of all variable and realize more clear classification of sample groups.

### 3.3 Multivariate modeling for the origin traceability of cherry

For more accurate multivariate modeling, outlier test must be first conducted for checking the abnormal values for every sample group using a 95% confidence interval test (α = 0.05) to evaluate the effects of possible data mistakes, the test results are as illustrated in Fig. 3. Obviously, though there are a few samples are out of 95% the confidence interval of sample group, e.g., S39, S68, S77, S78, S97, and S128, but their deviations from the confidence intervals of sample group are considerably small. In this study, most of isotopic data of samples (>99%) in each group are correct, a few of outliers can be overcome by various multivariate methods.

The origins of 153 cherry samples from five provinces in China were initially distinguished using unsupervised hierarchical cluster analysis (HCA). The resulting classification and the correlations between variables and samples were visually represented b a heat map, as depicted in Fig. 4A**.** HCA heat map, based on five stable isotope ratios and eight elemental contents of cherry samples, depicts the classifications of samples according to their origins by different colors, and presents the correlations and regional differences of variables. Generally, the classifier (i.e., origin axis) of cherry samples in HCA heat map shows that the origin labels of cherry samples are intricately mixed and cannot be distinctly clustered into five regions. Specifically, cherry samples from Shandong (green squares) and Liaoning (red squares) are nearly mis-classified into all other clusters of samples. The HCA heat map of variables shows that δ^15^N of cherry samples is of low correlation with cluster of samples, just slightly high expression in the cluster of samples from Shandong, δ^13^C, ^87^Sr/^86^Sr, δ^18^O, Mg, Ca, P, and K are all of high correlation in all clusters of samples, which indicate that less regional differences can be extracted from these variables of samples by HCA for classification. Relatively, δ^2^H and Se are obviously higher correlated with the cluster of sample from Sichuan than other provinces, but Fe, Na, and Zn are more correlated with Shandong, which indicates that more significant differences of these variables of cherry samples can be extracted by HCA and conduces to classify the geographical origins. Whereas, the variance information of five stable isotopic and eight multi-elemental variables are ineffective to differentiate all clusters of cherry samples from five provinces of China, just δ^2^H, Se, Fe, Na, and Zn can provide differential information to a certain extent. Consequently, the classifier for samples from different origins is malposed in this HCA heat map.

Then, PLS-DA modeling based on raw data matrix with the size of 153 × 13 (consisting of five stable isotope ratios and eight elemental contents of 153 cherry samples) was performed for origin traceability, the results were presented in Fig. 4B. The PLS-DA score plot of samples (Fig. 4**B**-1) projected on the first two latent variables (i.e., t1 and t2) shows the distribution of sample class, in which cherry samples from Shandong and Sichuan can be completely separated into two individual cluster regions. In addition, cherry samples from Liaoning can be generally verified even slightly overlapped with samples from Hebei and Shannxi. However, this 2D score plot of sample can't effectively verify the clusters of samples from Hebei and Shannxi, there are still heavy overlap between these two clusters of samples. To more clearly show the classification results of PLS-DA modeling, 3D score plot of samples projected on the first three latent variables (i.e., t1, t2 and t3) was illustrated, in which the overlapped samples from Hebei and Shannxi can be further separated by the dimension of the third latent variable (as shown in **Fig. S1 in supporting information**). As shown in the summary fit plot (Fig. 4**B**-2) of PLS-DA, the R^2^Y and Q^2^ parameters is used for evaluating the performance of this model, where R^2^Y describe the cumulative variance contribution rate based on the first few components and Q^2^ describe the prediction ability of the model's components. When the number of latent variables is increased to 4, the R^2^Y is 50.8%, Q^2^ reach a maximum value of 46.7%, indicating the model achieve the best fitting effect when the number of latent variables for PLSR was set at 4. The loadings plot (Fig. 4**B**-3) shows the contribution weights of variables, in which P, δ^13^C, δ^2^H, Zn, and K mainly contribute to the first latent variable (t1), Fe, Mg and δ^2^H mainly contributed to the second latent variable (t2). The VIP (variable importance of projection) plot of variables (Fig. 4**B**-4) indicate that P, K, Fe, δ^2^H, δ^13^C and Zn are the most important variables for the discrimination of the place of origin of cherry samples (VIP >1). The accumulative relative mean square error of crossing-validation (RMSEcv) predicted by the first four latent variables of PLS-DA has reached the lowest value of about 0.25 for all five cluster of samples (Fig. 4B-5), indicating the performance of PLS-DA model based on the first four latent variables is enough effective for the origin traceability of cherry samples from five provinces of China. The leave-one-out crossing-validation of PLS-DA are conducted, the discriminant accuracies are 96%, 96.48%, 100% for cherry samples from Liaoning, Shandong, Sichuan, but only 45.45% and 18.18% for cherry samples from Hebei and Shannxi (Table 2). Although supervised PLS-DA improves the clustering efficiency of cherry samples from different provinces relative to HCA, but it may still lead to big mis-discriminant probabilities for cherry from Hebei and Shannxi. The overall specificity and sensitivity of PLS-DA model were calculated as 0.67 and 0.73, the relatively small values indicate the deficiency for accurately identification, so other supervised method is needed for further enhancing the discriminant performance.

**Table 2:** Discriminant accuracies of 153 cherry samples from five provinces of China (Liaoning, Shandong, Hebei, Sichuan, and Shaanxi) using PLS-DA and LDA modeling and their performance parameters.

Modeling	Province	The number of samples	Discriminant accuracy	Liaoning	Shandong	Hebei	Sichuan	Shaanxi	Specificity	Sensitivity
**PLS-DA**	Liaoning	50	96.0%	48	2	0	0	0	0.67	0.73
	Shandong	66	98.5%	1	65	0	0	0		
	Hebei	11	45.5%	2	1	5	3	0		
	Sichuan	15	100%	0	0	0	15	0		
	Shaanxi	11	18.2%	6	0	0	3	2		
LDA	Liaoning	50	92.0%	46	0	4	0	0	0.87	0.93
	Shandong	66	98.5%	0	65	1	0	0		
	Hebei	11	90.9%	1	0	10	0	0		
	Sichuan	15	100%	0	0	0	15	0		
	Shaanxi	11	100%	0	0	0	0	11		

For achieving better classification and identification of the cherry samples from different provinces, LDA model is performed to reduce dimensionality and extract difference information of variables, the results were presented in Fig. 4C. The LDA score plot of samples (Fig. 4**C**-1) projected on the first two linear discriminant functions (Function 1 and 2) clearly exhibits more excellent clustering performance of samples relative to PLS-DA. Obviously, all samples from Shandong and Sichuan could be completely separated into two individual clusters, samples from Liaoning and Hebei can be also approximately divided, but samples from Shannxi are slightly overlapped with samples from Hebei and Liaoning. The classified indexes of cherry samples are calculated by four linear discriminant functions (Function 1 and Function 2) (Table 2), the discriminant accuracies for cherry samples from Hebei and Shannxi are significantly elevated to 90.9% and 100% by LDA from 45.45% and 18.18% in PLS-DA modeling, and reach 92.0%, 98.5% and 100% for cherry samples from Liaoning, Shandong, and Sichuan, respectively. The overall specificity and sensitivity of LDA model were calculated as 0.87 and 0.93, which indicate that the established LDA mode can significantly improve the clustering performance of cherry in China. In addition, the classification functions of cherry for five provinces of China can be inferred according to the following eqs. (2), (3), (4), (5), (6):

(2) $$Y_{\text{Liaoning}}\;\left[1\right]=-44.2^{*}\delta^{13}C+10.2^{*}\delta^{15}N-4.1^{*}\delta^{2}H-3.7^{*}\delta^{18}O+1.1\times 10^{4*87}\mathrm{Sr}/{}^{86}\mathrm{Sr}-0.4^{*}\mathrm{Na}+2.5^{*}\mathrm{Mg}+2.1^{*}P+0.4^{*}K+1.7^{*}\mathrm{Ca}+26.7^{*}\mathrm{Fe}+16.7^{*}\mathrm{Zn}+49.2^{*}\mathrm{Se}-5.9\times 10^{3}$$

(3) $$Y_{\text{Shandong}}\;\left[2\right]=-40.6^{*}\delta^{13}C+10.7^{*}\delta^{15}N-3.9^{*}\delta^{2}H-3.7^{*}\delta^{18}O+1.1\times 10^{4*87}\mathrm{Sr}/{}^{86}\mathrm{Sr}-0.3^{*}\mathrm{Na}+2.4^{*}\mathrm{Mg}+2.6^{*}P+0.4^{*}K+1.5^{*}\mathrm{Ca}+29.5^{*}\mathrm{Fe}+22.6^{*}\mathrm{Zn}+48.5^{*}\mathrm{Se}-6.0\times 10^{3}$$

(4) $$Y_{\text{Hebei}}\;\left[3\right]=-43.5^{*}\delta^{13}C+10.4^{*}\delta^{15}N-4.3^{*}\delta^{2}H-3.3^{*}\delta^{18}O+1.1\times 10^{4*87}\mathrm{Sr}/{}^{86}\mathrm{Sr}-0.3^{*}\mathrm{Na}+2.2^{*}\mathrm{Mg}+2.3^{*}P+0.3^{*}K+1.6^{*}\mathrm{Ca}+29.0^{*}\mathrm{Fe}+16.4^{*}\mathrm{Zn}+47.1^{*}\mathrm{Se}-5.8\times 10^{3}$$

(5) $$Y_{\text{Sichuan}}\;\left[4\right]=-46.4^{*}\delta^{13}C+11.9^{*}\delta^{15}N-4.8^{*}\delta^{2}H-3.3^{*}\delta^{18}O+1.2\times 10^{4*87}\mathrm{Sr}/{}^{86}\mathrm{Sr}-0.3^{*}\mathrm{Na}+2.1^{*}\mathrm{Mg}+2.0^{*}P+0.3^{*}K+1.7^{*}\mathrm{Ca}+29.5^{*}\mathrm{Fe}+16.0^{*}\mathrm{Zn}+51.4^{*}\mathrm{Se}-5.9\times 10^{3}$$

(6) $$Y_{\text{Shaanxi}}\;\left[5\right]=-43.7^{*}\delta^{13}C+12.3^{*}\delta^{15}N-4.6^{*}\delta^{2}H-3.3^{*}\delta^{18}O+1.2\times 10^{4*87}\mathrm{Sr}/{}^{86}\mathrm{Sr}-0.4^{*}\mathrm{Na}+2.3^{*}\mathrm{Mg}+2.0^{*}P+0.4^{*}K+1.8^{*}\mathrm{Ca}+27.7^{*}\mathrm{Fe}+18.8^{*}\mathrm{Zn}+50.7^{*}\mathrm{Se}-5.9\times 10^{3}$$

The ROC and AUC plot of variables (Fig. 4**C**-2) shows Mg, Na, δ^2^H, Ca and ^87^Sr/^86^Sr significantly contribute to the specificity and sensitivity of LDA model with large weight for the discrimination of the place of origin of cherry samples, their AUC values >0.5, so they can be regarded as the important contribution variables for this LDA discrimination.

By comparing these three multivariate methods in the origin traceability of cherry, unsupervised HCA was proved to be insufficient to differentiate the place of origin of cherry samples in China, the discriminant accuracies of PLS-DA model are still considerably low for some origins (only 45.45% and 18.18% for cherry samples from Hebei and Shannxi). Relatively, LDA can significantly improve the discriminant accuracies to the satisfactory levels exceeding 90% (92.0% for Liaoning, 98.5% for Shandong, 90.9% for Hebei, 100% for both Sichuan and Shaanxi). What is noteworthy is that the producing areas of China involved in this current origin traceability study are still too little (just few cherry origins in five provinces), it should be further enlarged in future study as the expansion of planting scales of cherry in China.

The importance and novelty of this work are mainly first exploring the feasibility of origin traceability of sweet cherry between geographical intervals of province-scale rather than country-scale, in which the selections of variables and modeling methods are more difficult for accurate discrimination. The study proves that the feasibility of origin discrimination of sweet cherry in different provinces of China using LDA modeling of stable isotopic and multi-elemental data together, and realize the discriminant accuracy higher than 90%. Therefore, this study will provide theoretical basic and technical support for the application of stable isotope and multi-element analysis to verify the place of origin of cherry products in Chinese market, ensure the origin label authenticity of high-price PGI cherry, and promote the development of cherry industry in China.

## 4 Conclusion

In this study, the stable isotopic and multi-elemental correlations of sweet cherry with the eco-system of its origin is deeply explained for stable isotopic fractionation and elemental accumulation mechanisms, which proves that the principles for origin traceability. δ^2^H, δ^18^O, δ^15^N, ^87^Sr/^86^Sr, Fe, Zn and Se values of cherry are highly correlated with its irrigation water and cultivated soil (*r* > 0.7). ANOVA analysis indicates that the regional variances of δ^2^H, δ^18^O and ^87^Sr/^86^Sr of cherry are significant (*P* < 0.05), making them suitable geographical indicators. 153 cherry samples from five provinces of China are discriminated by PLS-DA model yielding satisfactory accuracies for Liaoning (96.0%), Shandong (98.5%), and Sichuan (100%), but lower accuracies for Hebei (45.5%) and Shaanxi (18.2%). However, LDA significantly improves the discriminant accuracies of sweet cherry samples from all origins to the values exceeding 90%, thereby ensuring the effectiveness of this strategy for origin traceability. This strategy may provide a practical and efficient method for combating origin mislabeling and fraudulent conducts of cherry products in China, it is valuable for protecting PGI cherry products and ensuring food label authenticity.

## CRediT authorship contribution statement

**Shuanghui Wang:** Writing – original draft. **Piao Chen:** Writing – original draft, Validation. **Yuchao Liu:** Writing – original draft, Software, Resources, Project administration, Methodology, Investigation. **Chang Chen:** Visualization, Validation, Software, Data curation. **Jing Tian:** Visualization, Methodology, Investigation, Data curation, Conceptualization. **Zhi Liu:** Writing – review & editing, Supervision, Conceptualization. **Bin Li:** Writing – original draft, Visualization, Validation, Investigation, Data curation. **Xianxian Mei:** Visualization, Validation. **Youlan Chen:** Visualization, Validation. **Yue Zhang:** Resources, Investigation. **Chenghao Li:** Visualization, Validation, Software, Formal analysis, Data curation. **Can Xu:** Validation, Investigation, Formal analysis. **Hansheng Gong:** Writing – review & editing, Supervision, Funding acquisition, Conceptualization.

## Declaration of competing interest

The authors declare that they have no known competing financial interests or personal relationships that could have appeared to influence the work reported in this paper.

## Data Availability

Data will be made available on request.

## Appendix A Supplementary data

Supplementary material Image 1
