# Supplementary material for: Geographical origin traceability of sweet cherry (Prunus avium (L.) Moench) in China using stable isotope and multi-element analysis with multivariate modeling
Source: Food Chem X. 2024 May 16;23:101477. doi: 10.1016/j.fochx.2024.101477 (PMC11637194; doi:10.1016/j.fochx.2024.101477)
Supplement: Supplementary file 1 — Supplementary material [file mmc1.docx]

**Supporting information**

**Table S1** the instrument parameters of MC-ICP-MS for strontium isotope ratio (^87^Sr/^86^Sr) analysis of cherry.

| Instrument parameter | values or ranges |
| --- | --- |
| RF Power | 1299 W |
| RF Reflected Power | 3 W |
| Cooling air flow rate | 16.2 L/min |
| Auxiliary gas flow rate | 0.6 L/min |
| Carrier gas flow rate | 1.1 L/min |
| X-Position | 0.1 mm |
| Y-Position | 1.5 mm |
| Z-Position | 1.1 mm |
| Extraction lens | -1504.6 V |
| Focus lens | -570.6 V |
| Atomizer | Micromist.PFA Nebuliser |
| Injection Volume | 50 μL/min |
| Instrument Mass Resolution | 400 (Low) |
| Strontium Signal Sensitivity | 30 V/μg |
| Signal acquisition time | ca.10min (9 Blocks×10 cycles) |
| Integration time | 4.194 s/cycle |

**
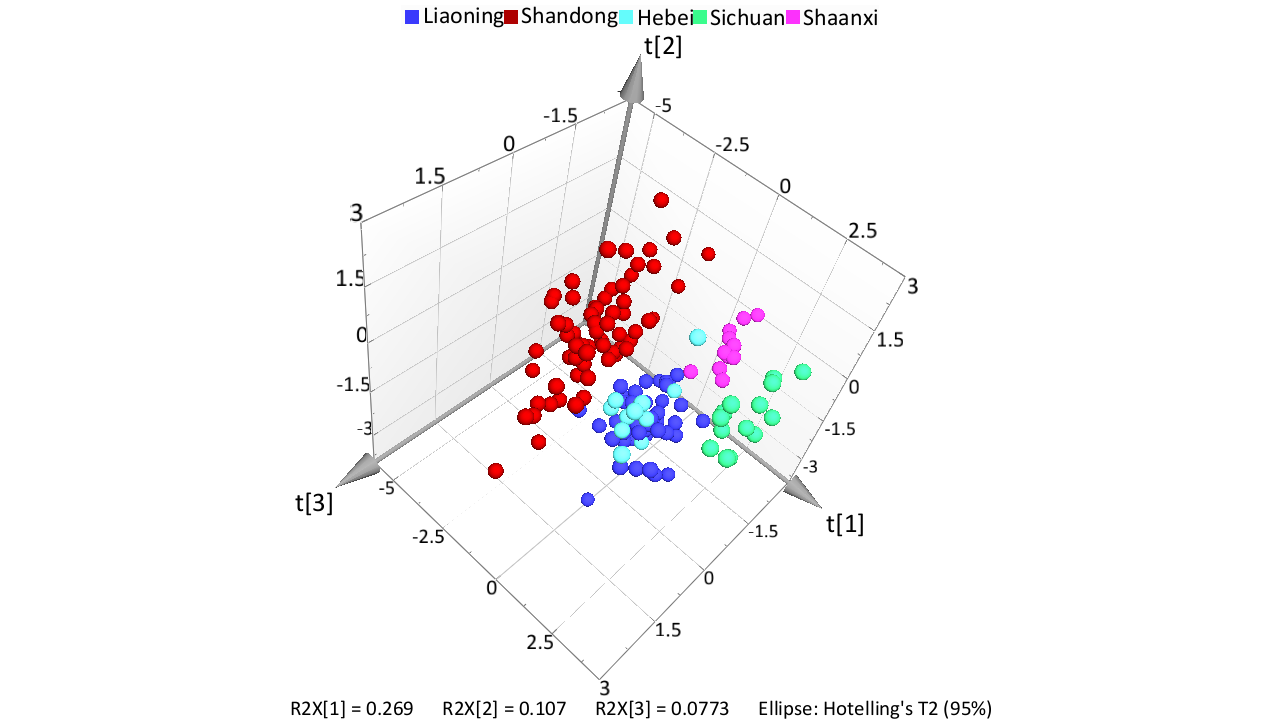
**

**Fig. S1** 3D PLS-DA score plot of cherry samples from five provinces of China (Liaoning, Shandong, Hebei, Sichuan, Shannxi).
